# The relationship between quality of life and compliance to a brace protocol in adolescents with idiopathic scoliosis: a comparative study

**DOI:** 10.1186/1471-2474-10-5

**Published:** 2009-01-14

**Authors:** LouAnn Rivett, Alan Rothberg, Aimee Stewart, Rowan Berkowitz

**Affiliations:** 1 Physiotherapy Department, School of Therapeutic Sciences, Faculty of Health Sciences, University of the Witwatersrand, Randburg, 2125, South Africa; 2 Sandton, Sunninghill and Morningside Clinics, Johannesburg, South Africa

## Abstract

**Background:**

Corrective bracing for adolescent idiopathic scoliosis (AIS) has favourable outcomes when patients are compliant. However, bracing may be a stressful and traumatic experience and compliance with a bracing protocol is likely to be dependent upon patients' physical, emotional and social wellbeing. The Brace Questionnaire (BrQ), a recently-developed, condition-specific tool to measure quality of life (QOL) has enabled clinicians to study relationships between QOL and compliance.

**Methods:**

The BrQ was administered to 31 AIS patients after a minimum of 1 year of wearing a brace. Subjects were 13–16 year old South African girls with Cobb angles of 25–40 degrees. Participants were divided into two groups according to their level of compliance with the bracing protocol. Brace Questionnaire sub- and total scores were compared between the two groups using the t-test for comparison of means.

**Results:**

Twenty participants were classified as compliant and 11 as non-compliant. Mean total BrQ scores (expressed as a percentage) were 83.7 for the compliant group and 64.4 for the non-compliant group (p < 0.001), and on analysis of the 8 domains that make up the BrQ, the compliant group scored significantly higher in the 6 domains that measured vitality and social, emotional and physical functioning.

**Conclusion:**

Poor compliance with a brace protocol is associated with poorer QOL, with non-compliant patients lacking vitality and functioning poorly physically, emotionally and socially. Quality of life for adolescents with idiopathic scoliosis may relate more to psychosocial coping mechanisms than to physical deformity and its consequences. It is important to establish whether remedial programmes are capable of addressing personal, group and family issues, improving QOL and promoting compliance.

## Background

In Adolescent Idiopathic Scoliosis (AIS) there is typically a three-dimensional deformity in which the spine deviates from the normal sagittal and coronal positions when standing upright, with the potential to develop into a fixed and unbalanced posture [1,2]. The aetiology is poorly understood, with AIS usually presenting in an otherwise healthy child [3]. Frequency is similar in boys and girls, however progression is more common and also more severe in girls. Bracing may be used to stabilise the condition if curves progress to 25 degrees and beyond [4]. The primary aims of conservative management of AIS are prevention of progression, improvement of pulmonary function, and treatment of pain [2,4].

High correction bracing has been shown to have favourable outcomes when the patient is compliant [5,6]. However, bracing is considered to be a traumatic experience which may leave lasting emotional scars [7-9]. Since AIS affects body configuration and is a condition that will impact on an adolescent over a number of years, it has the potential to adversely affect lifestyle and behaviour. The condition may itself precipitate social problems, with brace treatment further affecting self- and body image, interactions with others, overall quality of life (QOL), and generally being a stressful experience for patients [10-12]. Furthermore, wearing of the brace may be painful, and the brace may result in pressure areas [9]. As stated by Climent and Sanchez [13], clinical variables that may affect QOL include severity of the condition, skeletal maturity (Risser Sign), duration of brace treatment and degree of correction (conservative and/or surgical). However, the extent to which all these factors influence a particular patient's ability to benefit from the brace will depend on his/her physical, emotional and social wellbeing. In fact, psychological issues alone have been the cause for a lack of compliance to a brace protocol [14], and approximately 9% of girls will discontinue wearing a brace because of psychological distress [15]. Clinicians therefore need to be aware of factors that affect social wellbeing, and how these factors relate to psychosocial functioning [16]. This having been said, there is nevertheless considerable debate as to whether patients with scoliosis treated with braces experience a poor QOL [10]. The Brace Questionnaire (BrQ) is a condition-specific questionnaire that has been developed, validated and translated into English by Vasiliadis et al [10]. It is specifically designed to assess QOL in children with AIS who are treated conservatively with a brace [See Additional file 1: Brace Questionnaire]. As reported by the developers of the BrQ, patients with moderate or severe scoliosis have poorer scores than those with mild scoliosis, and the tool is sensitive to changes over time (measuring improvement or deterioration of QOL according to correction or worsening of the physical condition)[17]. Thus the purpose of this study was to compare the QOL in patients who comply with a brace protocol with those who do not.

## Methods

This assessment of QOL in brace-compliant and non-compliant subjects is part of a larger study that has as its main objective the assessment of outcomes in AIS patients treated with appropriate bracing and individualised exercise programmes for the appropriate length of time (as determined by the Risser sign). All subjects are being treated in a private practice by L.R. following referral by various orthopaedic surgeons, who prescribed full-time wearing of the brace (23 hours per day). The sample for this study into compliance was a convenience sample that included all girls within the practice who had a diagnosis of AIS, were 13–16 years of age, had not been surgically-treated and were not yet eligible for weaning from their brace. Extent of the deformity as measured by Cobb angles was between 25 and 40 degrees, and all girls were eligible for intervention that included bracing as per SOSORT (Society on Scoliosis Orthopedic and Rehabilitation Treatment) guidelines [4]. Apical vertebral rotation (Pedriolle method) ranged between 5 and 30 degrees(mean of 17 degrees), angle of trunk inclination using a scoliometer ranged 5 to 17 degrees (mean of 10 degrees) and Risser sign 0 to 3 (All Risser 3 patients had Cobb angles greater than 35 degrees). Patients were fitted with the Rigo System Cheneau (RSC) Brace, a device that addresses a wide range of curves and AIS-related deformities [18]. Informed consent was obtained from all subjects and their parents. Ethical clearance was obtained from the Committee for Research on Human Subjects at the University of the Witwatersrand (Reference M060702).

The BrQ, as developed and translated by Vasiliadis et al [10] was administered to 31 subjects after a minimum of one year of wearing the RSC brace and before initiation of weaning from the device. The BrQ was administered in the private practice of LR during a routine patient visit, with the investigator available throughout for any explanation or clarification required by study participants. Applicable to subjects between 9 and 18 years of age, the BrQ takes 10–15 minutes to complete. It is comprised of 34 questions in 8 domains: general health perception, physical functioning, emotional functioning, self esteem and aesthetics, vitality, school activity, bodily pain and social functioning. Each question is scored, domain sub-scores are calculated, and a total BrQ score is obtained. A percentage score is then calculated. A minimum score of 20 is theoretically possible and a maximum score is 100. A higher score indicates a better QOL.

Subjects were divided into two groups on the basis of their compliance histories. For the purpose of this study, compliance was defined as wearing of the brace for 20–23 hours per day and compliance to a prescribed exercise routine, ideally carried out at least four times per week. Non-compliant subjects wore the brace for fewer than 20 hours per day and exercised less than four times per week. Actual hours of brace wearing per day and number of exercise days per week were recorded in a diary issued to each subject. The diary was filled in every day, was regularly checked by parents, and was reviewed by L.R. once per month, at which time the contents were confirmed in a private meeting with parents.

The individual BrQ scores of the compliant and non- compliant groups were then analysed. Descriptive statistics for each group, differences between the groups, correlations and regression analysis were computed using TexaSoft's WINKS SDA Professional Edition, version 6. Statistical significance was accepted with p-values <0.05.

## Results

Of the 31 patients who completed the BrQ, 20 were classified as compliant and 11 as non-compliant. Mean ages (± SD) of the compliant and non-compliant groups were 14.6(± 1.8) and 15.7(± 2.2) years respectively (difference not significant). Compliant subjects wore the brace for 21.4(± 0.9) hours per day while those in the non-compliant group admitted to wearing the brace for only 14.4(± 7.6) hours per day (p < 0.02). This difference is expected to be significantly different because of the *a priori *separation of the groups on the basis of the hours per day in the brace; however the magnitude of the difference (21.4 vs. 14.4 hours per day) suggests that the non-compliant group mainly wore the brace while at home and not when exposed to peers, school etc. While there are several factors that influence compliance, it is also possible that **duration **of brace-wearing adds to the problem i.e. the longer the experience of wearing the brace the greater the reluctance to wear it. In this regard there was a trend towards the compliant group having been in the brace for a shorter period of time (10.4(± 10.4) vs. 16.8(± 13.5) months; difference not significant).

**Figure 1:**
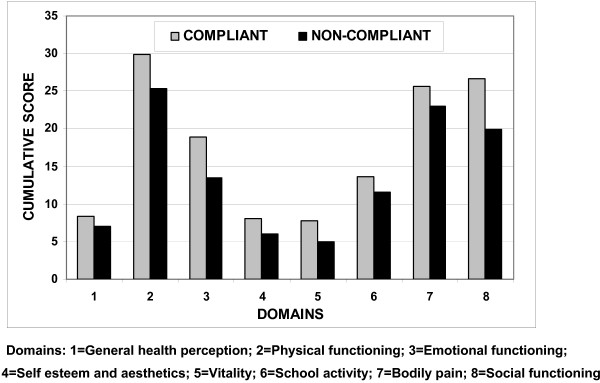
**Distribution of mean sub-scores for compliant and non-compliant groups in each of 8 domains of the Brace Questionnaire **[10].

Figure 1 shows the mean sub-scores in each of the 8 domains covered by the questionnaire. These scores differ in magnitude because the 34 questions are not evenly distributed between the domains i.e. some have only two questions in the particular domain while others have up to seven. What is clear from the figure is that across the board the scores in the non-compliant group are lower than those of the compliant patients. Overall scores for the two groups (i.e. sum of all domain scores with correction to obtain a percentage) were 83.7(± 8.3) for compliant patients vs. 64.4(± 10.6) for those who did not comply (p < 0.001).

Table 1 gives further detail of the differences between the groups. It is noteworthy that non-compliant subjects do not regard themselves as being in poor general health or experiencing bodily pain (differences between the groups not significant for these two domains), but they clearly lack vitality, have low self esteem, and physical, emotional and social function is at a significantly lower level.

**Table 1:** Detailed comparison of scores in each of the 8 domains for compliant and non-compliant groups

Domain	Compliant (n = 20)	Non-Compliant (n = 11)	Difference (p-value)
**General Health**	8.4 ± 1.6	7.0 ± 2.3	Not significant

**Physical Functioning**	29.9 ± 2.5	25.3 ± 4.3	0.006

**Emotional Functioning**	18.9 ± 3.7	13.5 ± 4.9	0.006

**Self-Esteem and Aesthetics**	8.0 ± 1.1	6.0 ± 1.9	0.008

**Vitality**	7.8 ± 1.2	5.0 ± 1.5	<0.001

**School Activity**	13.6 ± 1.4	11.6 ± 2.3	0.02

**Bodily Pain**	25.7 ± 3.5	23.0 ± 3.7	Not significant

**Social Functioning**	26.7 ± 3.5	19.9 ± 6.0	0.004

Regression analysis of the data, with compliance or non-compliance as the dependent variable, indicates that the responses to the 34 questions in the eight domains of the BrQ account for some 61% of the variance in compliance, with differences in Vitality (the major contributor) and Social and Emotional Functioning together accounting for 90% of that variance.

There was no correlation between total BrQ score and severity of scoliosis as measured by Cobb angle for the 31 subjects analysed (Pearson's correlation coefficient 0.255).

## Discussion

Since AIS affects body configuration and is a condition that will impact on an adolescent, at least over a number of years, AIS on its own has the potential to adversely affect lifestyle and behaviour. Bracing, which is an important intervention during the years preceding skeletal maturity, has been shown to have favourable physical and structural outcomes, with a direct relationship existing between outcome and patient compliance with the treatment [5,6]. However, it should be borne in mind that brace wearing for some patients is a traumatic experience that is superimposed on the psychosocial stresses of the underlying physical condition and may leave lasting emotional scars [7-9]. The combination of AIS as a disease and intervention (brace application) may therefore conspire to adversely affect self- and body image, interactions with others and overall QOL; in general being a stressful experience for patients [10-12].

Given that there is a correlation between physico-structural outcome and compliance with a bracing protocol [5,6], it is important for all members of the clinical team dealing with AIS to be aware of factors that will reduce the amount of time that patients wear the brace, either in terms of hours per day, or months of use. In this regard, psychological issues have previously been shown to influence compliance in terms of brace wear [14], with approximately 9% of girls discontinuing brace wearing as a result of being stressed [15].

In this study involving 31 subjects it was shown that if compliance is defined as brace wearing for = 20 hours per day, then approximately one-third of patients did not comply with the protocol. Vandal et al showed that self-reporting overestimated duration of brace-wearing when compared against time as physically measured by a device attached to the brace [19]. However, since such a device is not available in South Africa, it was important to verify compliance by monitoring it carefully and thoroughly. This was achieved by the patient dilligently completiing a diary daily, which was routinely verified by parents and regularly monitored by one of the investigators. Analysis revealed that while there was a statistically insignificant trend towards non-compliance being associated with longer exposure to the brace (i.e. months since initiation), it was the QOL as measured by the BrQ that correlated with brace-wearing behaviour. To some extent these findings are inconsistent with those of Beka et al [20] and Ungwonali et al [21] who found that QOL was not affected by brace wearing; however the QOL measures used by those authors, particularly in the latter study, were generic, and not condition-specific as was the case in this study. It is important to note that subjects who were non-compliant in this study did not see themselves as being sickly or in pain, but they had poor self image and self esteem, and functioned poorly in the physical, emotional and social domains. This finding that certain components of the BrQ may be more useful than the overall score is in keeping with results published by Vasiliadis et al [22]. That group also showed that BrQ scores may be related to the degree of deformity [10]; however this was not found in this study i.e. there was no indication that poorer QOL as measured by the BrQ was related to (higher) degree of deformity, and it would therefore appear that QOL issues may be related more to psychosocial coping mechanisms than to physical deformity and its consequences.

There have been several studies that have delved into other aspects of stress in adolescents with idiopathic scoliosis. For example, AIS-related anxiety has been considered to be the result of not knowing whether the spinal deformity and its symptoms will progress [14]. In a large study involving 685 adolescents, Payne et al showed that girls with scoliosis were 55% more likely to have suicidal thoughts, and three times more likely to consume alcohol after school than girls without scoliosis [23]. Boys with scoliosis were 95% more prone to alcohol consumption and ten times more inclined to have suicidal thoughts [23]. These latter studies speak to both the causes and consequences of the QOL issues identified in the group of patients under review here, and raise the important question as to how one should act to mitigate the problems. Support for AIS patients in the form of psychological group sessions and individual sessions has been shown to have an effect in preventing psychosocial impairment [24] and should clearly be considered for inclusion in holistic management plans. Implicit in such plans is the need for all members of the healthcare team to be aligned in their attitudes towards the treatment programme since scepticism on the part of any team member will quickly be sensed by the patient and undermine the process. Ultimately the way in which an individual patient with AIS responds to the condition may also be a function of the home and family environment, implying that group and/or individual therapy may be inadequate. In this regard, data are currently being collected for the patients under discussion here as well as for other patients, and will be analysed and presented in a future publication.

## Conclusion

Recognising that results of this study cannot be generalised because of the relatively small sample size, and notwithstanding the reliance on patient reporting to measure compliance, the findings are nevertheless of interest and probably also of importance. Poor compliance to a brace protocol is associated with poorer QOL, with non-compliant patients lacking vitality and functioning poorly physically, emotionally and socially. Quality of life for adolescents with idiopathic scoliosis may relate more to psychosocial coping mechanisms than to physical deformity and its consequences. It is important to establish whether remedial programmes are capable of addressing personal, group and family issues, improving QOL and promoting compliance.

## Competing interests

The authors declare that they have no competing interests.

## Authors' contributions

LR designed the study, applied exercise routines, administered brace questionnaires, acquired the data, analysed and interpreted the data, drafted the manuscript, and gives final approval of the version to be published. AR interpreted the data, revised the manuscript, and gives final approval of the version to be published. AS assisted with study design, and drafting of manuscript, and gives final approval of version to be published. RB assisted with study design, collection of data, and gives final approval of version to be published.

## Pre-publication history

The pre-publication history for this paper can be accessed here:



## Supplementary Material

Additional file 1 **The Brace Questionnaire.** The English translation of the Brace Questionnaire and its 34 items. Click here for file
